# Recent intensification of winter haze in China linked to foreign emissions and meteorology

**DOI:** 10.1038/s41598-018-20437-7

**Published:** 2018-02-01

**Authors:** Yang Yang, Hailong Wang, Steven J. Smith, Rudong Zhang, Sijia Lou, Yun Qian, Po-Lun Ma, Philip J. Rasch

**Affiliations:** 1 Atmospheric Sciences and Global Change Division, Pacific Northwest National Laboratory, Richland, Washington, USA; 2 Joint Global Change Research Institute, Pacific Northwest National Laboratory, College Park, Maryland USA

## Abstract

Wintertime aerosol pollution in the North China Plain has increased over the past several decades as anthropogenic emissions in China have increased, and has dramatically escalated since the beginning of the 21^st^ century, but the causes and their quantitative attributions remain unclear. Here we use an aerosol source tagging capability implemented in a global aerosol-climate model to assess long-term trends of PM_2.5_ (particulate matter less than 2.5 μm in diameter) in the North China Plain. Our analysis suggests that the impact of China’s increasing domestic emissions on PM_2.5_ concentrations over the last two decades of 20^th^ century was partially offset (13%) by decreasing foreign emission over this period. As foreign emissions stabilized after 2000, their counteracting effect almost disappeared, uncovering the impact of China’s increasing domestic emissions that had been partially offset in previous years by reductions in foreign emissions. A slowdown in the impact from foreign emission reductions together with weakening winds explain 25% of the increased PM_2.5_ trend over 2000–2014 as compared to 1980–2000. Further reductions in foreign emissions are not expected to relieve China’s pollution in the future. Reducing local emissions is the most certain way to improve future air quality in the North China Plain.

## Introduction

China has suffered from many severe haze episodes in recent decades^1,2^. One of the most densely populated regions in the world, the North China Plain is the most polluted region of the country^3^. Due to economic growth and rapid urbanization, air quality in the North China Plain is deteriorating, characterized by high PM_2.5_ concentrations^4^. In January 2013, Beijing, the country’s capital and major city in the North China Plain, was hit by severe and persistent haze events^5^, with the maximum daily PM_2.5_ concentration exceeding 500 μg m^−3^, leading to Beijing’s first haze orange alert in history^6^.

Exposure to these fine particles harms respiratory and cardiovascular systems, causing morbidity and mortality^7–9^. The high PM_2.5_ concentrations found during haze episodes also drive atmospheric visibility decreases, endangering air transportation and road traffic, and consequently imposing an additional adverse effect on economic activities^10,11^. Through long-range transport, these aerosols can also reach distant and remote areas, resulting in global impacts on air quality and climate change^12,13^.

Winter haze events in the North China Plain have been increasing over past decades^6,14^. During extreme winter haze episodes, more aerosol particles are produced and accumulated in the boundary layer associated with stagnant meteorological conditions^15,16^, leading to high PM_2.5_ concentrations. Recent studies indicated that the weakening of wind speed plays an important role in the long-term increase of pollutants^17,18^. The intensified China winter haze has also been tied to the East Asian monsoon^19–21^, Arctic sea ice^22,23^ and Pacific Decadal Oscillation^24^ through climate change^25^. In addition, dust-wind interactions^26^ and absorbing aerosol-planetary boundary layer interactions^27,28^ have been found to affect PM_2.5_ concentrations during China winter haze events.

Although meteorological conditions play an important role in mixing and advecting pollutants, increasing local aerosol emission from human activities is generally considered to be the dominant cause of increasing wintertime haze over China in past decades^29^. The frequency of winter haze days in China has shown steep increases since the beginning of the 21^st^ century^14^, which is also attributed to increasing anthropogenic emissions^30^. However, aerosol pollution is not just a local issue. Aerosols in the North China Plain partly arise from emissions from other regions of China and transboundary transport from foreign countries/regions (e.g., South Asia, Central Asia, Russia, and Europe)^31^. As anthropogenic emissions in China have been climbing, foreign regions (e.g., Europe and North America) have been significantly reducing their emissions during the past decades^32^. The reductions in foreign emissions may have led to a decrease in their contributions to aerosols in the North China Plain and partly offset the increase of aerosols from domestic emissions. However, the quantitative roles of changes in domestic and foreign emissions in decadal variations of aerosol concentrations in the North China Plain are not well understood. Emissions from both China and foreign countries are projected to decrease^33^ in the future and the relative importance of these emissions to future changes of wintertime air quality in China remains unclear.

Here, we examine the variations of wintertime PM_2.5_ concentrations on decadal timescales for the North China Plain over 1980–2014 to quantify and attribute aerosol trends due to emissions and meteorology from different regions using an aerosol tagging technique. Our goal is to quantitatively examine the factors influencing decadal variations of aerosols and causes of the steep increase in aerosol pollution in the North China Plain since the beginning of the 21^st^ century.

## Results

### Aerosol decadal variation and source attribution

**Figure 1:**
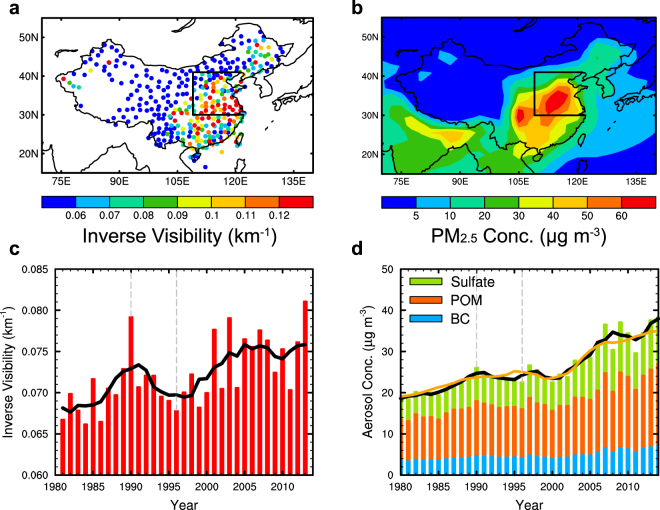
(**a**) Observed inverse value of atmospheric visibility (km^−1^) and (**b**) model simulated PM_2.5_ concentrations (μg m^−3^) averaged in December 2012 and January-February 2013. Time series of DJF mean (**c**) inverse visibility and (**d**) simulated near-surface aerosol concentration (bars) averaged over the North China Plain (109°E–east border, 30–41°N, boxed area in **a** and **b**). The inverse of visibility is calculated as the reciprocal of regional averaged visibility in the North China Plain. Black lines represent five-year moving average. Orange line in panel d represents normalized PM_2.5_ concentrations (see methods). Maps were generated by NCAR Command Language (NCL) version 6.4.0 (Boulder, Colorado: UCAR/NCAR/CISL/TDD, 10.5065/D6WD3XH5).

There are long-term records for atmospheric visibility (visual range) over decades in China that have been widely used in studies of aerosol variation^23,34,35^. The inverse value of visibility (see methods), which is closely related to light extinction (km^−1^), is a good measure of light attenuation caused by aerosols. Figure 1a shows the spatial distribution of December-January-February (DJF) mean observed inverse of atmospheric visibility over China in 2013, when several severe haze events occurred. High values of inverse visibility are evident over eastern China, and especially the North China Plain, indicating high aerosol concentrations over these regions. The CESM (Community Earth System Model) simulated DJF mean PM_2.5_ concentrations over China in 2013 (Fig. 1b) have a similar spatial distribution, with maximum seasonal mean PM_2.5_ concentrations larger than 60 μg m^−3^ in the North China Plain. Averaged over the North China Plain, both the observed inverse visibility and modeled PM_2.5_ concentration (sum of dry sulfate, BC (black carbon) and POM (primary organic matter) in this study, see Methods) show increasing trends over 1980–1990, followed by decreasing trends between 1990–1996 and an increasing trend in 1996–2014 (Fig. 1c,d). The temporal correlation coefficient (+0.70) between the two is statistically significant with 95% confidence. These suggest that variations of wintertime aerosol pollution on decadal timescales in the North China Plain can be approximately reproduced in the model.

**Figure 2:**
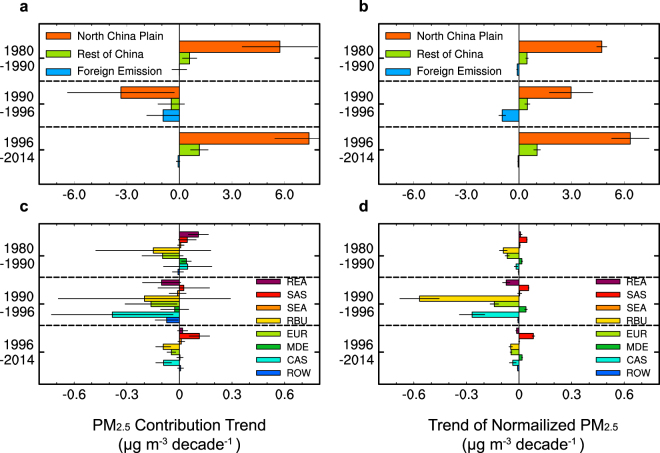
Linear trends (μg m^−3^ decade^−1^) of (**a**) the model simulated and (**b**) normalized DJF mean PM_2.5_ concentrations over the North China Plain contributed by the North China Plain, rest of China and foreign emissions for the three time periods, and (**c**,**d**) decomposed foreign contributions by tagged source regions. Black lines on the bars represent 95% confidence intervals of the linear regression. Source regions include the North China Plain (NTC), Southern China (STC), Southwestern China (SWC), Central-Western China (CWC), Northeastern China (NEC), Himalayas and Tibetan Plateau (HTP), rest of East Asia (REA), South Asia (SAS), Southeast Asia (SEA), Russia/Belarus/Ukraine (RBU), Europe (EUR), the Middle East (MDE), Central Asia (CAS) and rest of the World (ROW).

Tagging aerosol sources facilitates attribution to different source regions. Local emission changes explain the increasing trend of DJF mean PM_2.5_ over the North China Plain (Fig. 2a) during 1980–1990 of 5.7 (±2.1) μg m^−3^ decade^−1^, dominating the trend of 6.3 (±2.3) μg m^−3^ decade^−1^ from all sources (both China and foreign emissions). Changes in foreign emissions do not contribute to a discernable trend of DJF mean PM_2.5_ concentration in the North China Plain over this period because the negative trend associated with emission changes from Russia/Belarus/Ukraine and Europe (especially from western Europe) counters the positive trend from other foreign source regions (Fig. 2c).

Over 1990–1996, the contributions to DJF mean PM_2.5_ concentration in the North China Plain from almost all the tagged source regions decrease, leading to an overall trend of −4.7 (±2.8) μg m^−3^ decade^−1^, which can be decomposed to −3.3 (±3.0), −0.5 (±0.8) and −0.9 (±0.9) μg m^−3^ decade^−1^ from North China Plain, rest of China and foreign emissions, respectively.

During 1996–2014, the local contribution of the North China Plain increases at a rate of 7.4 (±1.9) μg m^−3^ decade^−1^, followed by 1.1 (±0.5) μg m^−3^ decade^−1^ contributed by emissions from rest of China. The combination of increasing trend of South Asia contribution and decreasing trends of Russia/Belarus/Ukraine, Europe and Central Asia leads to a very weak net trend of −0.1 (±0.1) μg m^−3^ decade^−1^ from foreign emissions.

Sulfate, POM and BC concentrations share similar increasing and decreasing trends with PM_2.5_ in DJF over the North China Plain between 1980 and 2014 (Supplementary Fig. S2). Sulfate is responsible for 34–40% of the decadal trends for the analyzed three periods. POM and BC account for 45–47% and 15–19% of the decadal trends of PM_2.5_, respectively. Foreign emissions have a much larger influence on the sulfate concentration changes than on POM and BC, because SO_2_ (the precursor gas of sulfate) from industrial and power-plant sources is evenly emitted at altitudes between 100–300 meters above the surface^36^, unlike POM and BC. The elevated emissions favor further lifting to the free troposphere and long-range transport, and also further contributing to the already less efficient scavenging of gaseous SO_2_ compared to BC and POM particles. Over the North China Plain, local emissions account for more than 90% of near-surface SO_2_ concentrations in DJF during 1980–2014 (figure not shown), much higher than the 60–70% local contribution fraction for particulate sulfate concentrations. During 1990–1996, the magnitude of decreasing trend of foreign contributions to the North China Plain sulfate concentration (−0.9 μg m^−3^ decade^−1^) exceeded the decrease in local emission contribution (−0.8 μg m^−3^ decade^−1^).

### Contributions from meteorology and domestic and foreign emissions

**Figure 3:**
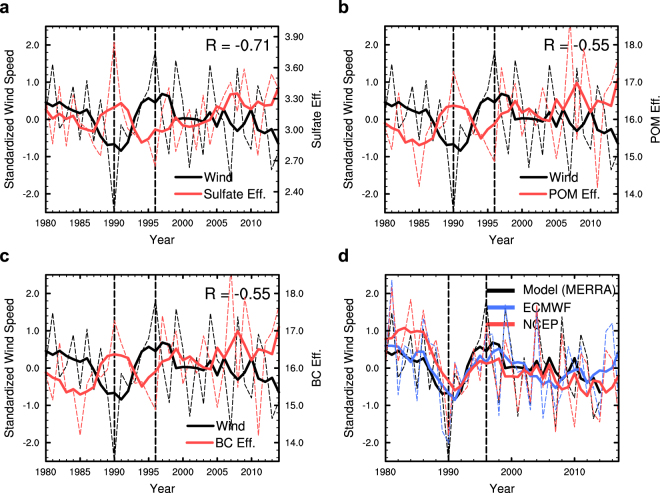
Time series of standardized regional mean wind speed (black dashed lines, unitless) and local concentration efficiency of (**a**) sulfate, (**b**) POM and (**c**) BC (red dashed lines, μg m^−3^ Tg S^−1^ for sulfate and μg m^−3^ Tg C^−1^ for POM and BC) for the North China Plain emission. (**d**) Standardized wind speed derived from MERRA reanalysis used for wind nudging in this study, ECMWF reanalysis and NCEP/NCAR reanalysis data. Solid lines represent five-year moving averages. Temporal correlation coefficient (R) between unsmoothed aerosol concentration efficiency and standardized wind speed is shown at the top-right corner of panel a, b and c.

It is interesting that DJF mean emissions of SO_2_, BC and POM from the North China Plain increased over 1990–1996 (Supplementary Fig. S3), while inverse visibility and modeled aerosol concentrations decreased (Fig. 1c,d). Tagging indicates that aerosol concentrations in the North China Plain produced by local emissions actually decreased with time, hinting that other processes are also influencing aerosol amounts. Aerosol concentration efficiency, defined as the source contribution to aerosol concentration over the North China Plain divided by total emission from the source region under consideration, can be used to quantify the impact of other processes (e.g., accumulation by stagnation, removal by precipitation, transport by winds) on the relationship between aerosol emission and concentration (see Methods). All the sulfate, POM and BC concentration efficiencies from the North China Plain local emissions show decreasing trends over 1990–1996 and slightly increasing trends over 1996–2014 (Fig. 3a–c, red lines). For fixed emissions with time, changes in concentration efficiencies would produce proportional changes in aerosol concentrations. The decrease in aerosol concentration efficiency explains the decreasing PM_2.5_ concentration from local emissions over 1990–1996 (Fig. 2a). The increasing concentration efficiencies after 1996 also contribute to the increasing aerosol pollution in the recent two decades.

Aerosol concentration efficiency is mainly influenced by meteorological factors. Low wind speed and temperature inversions often cause severe aerosol pollution events^23^. A useful metric is the standardized wind speed, defined as

1 $$Standardized\,W{S}_{i}=(W{S}_{i}-W{S}_{mean})/W{S}_{mean}$$

where ${{WS}}_{i}$ and ${{WS}}_{{mean}}$ represent regional mean wind speed at 925 hPa in DJF of year $i$ during 1980–2014 and mean value over the entire time period, respectively. The standardized wind speed over the North China Plain, black lines in Fig. 3a–c, has a strong negative correlation with aerosol concentration efficiencies over the North China Plain (i.e., −0.71 for sulfate, −0.55 for POM and BC). It increased over 1990–1996, favoring ventilation of aerosols and leading to decreasing PM_2.5_ concentrations. The standardized wind speed then decreased over 1996–2014, accumulating pollutants in the atmospheric boundary layer and partly accounting for the increasing PM_2.5_ concentrations. In addition to the MERRA reanalysis used in this study, a similar time variation of standardized wind speed also exists in other meteorological reanalysis data (Fig. 3d). Although temperature inversion can also be important for building up pollutions in the boundary layer, a pollution potential index^23^ defined by combining wind speed and temperature inversion does not give a much stronger (even weaker for sulfate) correlation with aerosol concentration efficiencies (Supplementary Fig. S4) than the wind speed alone during winter, indicating that wind speed is more important than the temperature inversion in driving decadal variations of wintertime aerosol concentrations over the North China Plain. While precipitation can be important in determining aerosol lifetime and abundance, it seldom rains over the North China Plain in winter. A recent study found that precipitation exerts negligible influence on interannual and decadal variations in wintertime PM_2.5_ concentrations in the North China Plain^18^.

One can isolate meteorological changes from emission changes by normalizing the aerosol concentrations by aerosol concentration efficiencies for each source region (See Methods), which allows assessment of the relative importance of domestic and foreign emissions on the aerosol trends. The normalized PM_2.5_ concentrations (orange line in Fig. 1d) show the same increasing pattern over 1980–2014 as the original modeled concentrations for the long-term trend. During 1990–1996, however, the normalized PM_2.5_ concentration increased from 23.7 μg m^−3^ in 1990 to 25.0 μg m^−3^ in 1996, proportional to local emission trends for this time period (from 1.44 to 1.73 Tg S for SO_2_, 0.72 to 0.75 Tg C for POM, and 0.25 to 0.26 Tg C for BC) (Supplementary Fig. S3).

Figure 2b quantifies the contribution to the overall trend of normalized PM_2.5_ concentrations from the North China Plain, rest of China and foreign emissions over the three time periods. After the influence of meteorology is removed, the normalized PM_2.5_ concentrations contributed by China emissions show increasing trends in all three time periods. Local emission changes from the North China Plain produce the largest trends in normalized PM_2.5_, with values of 3.0~6.3 μg m^−3^ decade^−1^. Emissions from the rest of China lead to 0.5~1.0 μg m^−3^ decade^−1^ increases. The increasing trend over 1980–1990 and 1996–2014, attributed to China emissions, is smaller in the normalized concentrations that in the original modeled PM_2.5_ concentrations (Fig. 2a). It indicates that besides the increasing anthropogenic emissions in China, the decadal variability of meteorology (e.g., weakening of wind speed) is partly responsible for changes in wintertime PM_2.5_ concentrations in the North China Plain. During 1990–1996, the meteorology effect outweighs the changes in China emissions, leading to a net decreasing trend of modeled PM_2.5_. Foreign emission contributions to the trend remain negative (around −1.0 μg m^−3^ decade^−1^) for both modeled and normalized PM_2.5_ concentrations. Emission reductions from the important upwind source regions (e.g. Russia/Belarus/Ukraine, Europe and Central Asia, Supplementary Fig. S1) have stabilized in the recent two decades (Supplementary Fig. S3). Therefore, foreign contributions did not have a large contribution to the trend in 1996–2014 as compared to 1990–1996. The contribution from South Asia emissions increased at a rate of 0.1 μg m^−3^ decade^−1^, offsetting the decreasing contributions from other foreign source regions, leading to a negligible net trend in foreign contributions over 1996–2014.

**Figure 4:**
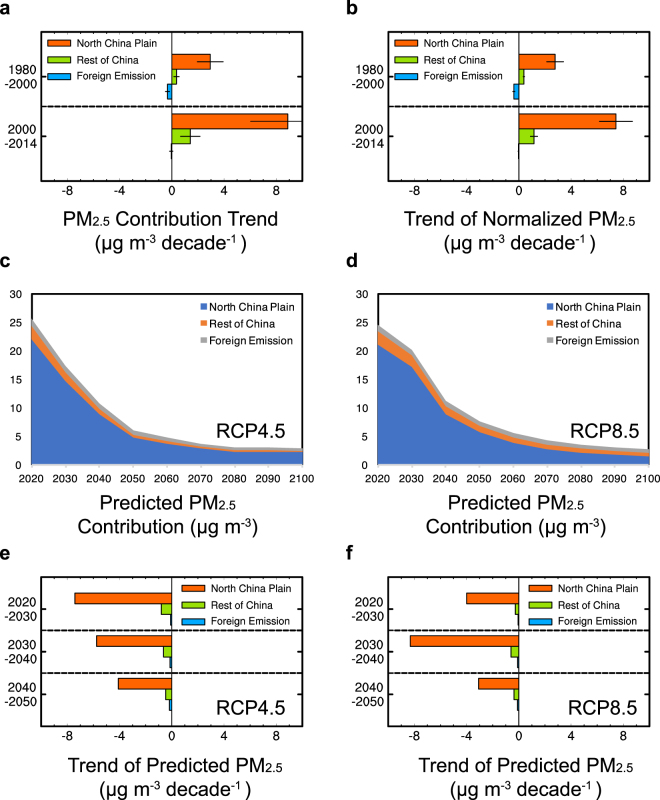
Linear trends (μg m^−3^ decade^−1^) of (**a**) the model simulated and (**b**) normalized DJF mean PM_2.5_ concentrations over the North China Plain contributed by the North China Plain, rest of China and foreign emissions for 1980–2000 and 2000–2014. Time series of predicted PM_2.5_ concentrations (μg m^−3^), averaged over the North China Plain, contributed by the North China Plain, rest of China and foreign emissions under (**c**) RCP4.5 and (**d**) RCP8.5 emission scenarios. Linear trends of predicted PM_2.5_ concentrations (μg m^−3^ decade^−1^) over 2020–2030, 2030–2040 and 2040–2050 contributed by the North China Plain, rest of China and foreign emissions under (**e**) RCP4.5 and (**f**) RCP8.5.

Since the aerosol pollution in China showed steep increases from the beginning of the 21^st^ century (Fig. 1d, 2001–2014), we combine the first two short periods (1980–2000) that had weak trends to quantitatively examine the relative importance of factors during the last two decades of the 20^th^ century in contrast to trends over 2001–2014. Modeled PM_2.5_ concentrations in the North China Plain from all sources increased about 3 times faster during 2000–2014 than 1980–2000 (Fig. 4a). The larger trend in 2000–2014 compared to 1980–2000 can be decomposed into contributions from emissions and meteorology (see methods). If the influence of meteorology is removed (Fig. 4b), then PM_2.5_ would have produced a smaller difference of trends between post- and pre-2000 (5.8 μg m^−3^ decade^−1^), compared to the total difference of trends (7.2 μg m^−3^ decade^−1^). It indicates that 81% of the total difference of trends arises from changes in emissions. The remaining balance (19%) is due to meteorological variations during those periods. Changes in domestic emissions (the North China Plain + rest of China) explain 75% of change of modeled PM_2.5_ trends pre- and post-2000. The PM_2.5_ trend from reductions in foreign emission between 1980–2000 (−0.4 μg m^−3^ decade^−1^) offset 13% of the increasing trend of modeled PM_2.5_ concentration in the North China Plain during the same period (3.0 μg m^−3^ decade^−1^). This dampening effect from reductions in foreign emissions mainly occurred during 1990–1996. As foreign emissions became stabilized, their counteracting effect disappeared in 2000–2014, in effect, uncovering additional pollution increases from domestic emissions that were hidden by changing foreign emissions pre-2000. It explains 6% of the change in trends of PM_2.5_ concentration in the North China Plain between pre- and post-2000 periods.

When we choose the baseline period when meteorology played a very important role, and calculate trend difference between 1990–1996 and 1996–2014 periods, the contribution of meteorology to the change in PM_2.5_ trends can be up to 64%, highlighting the importance of meteorology in influencing aerosol concentrations over relatively short time periods. The foreign emissions are responsible for 8% of the additional increasing trend in 1996–2014 compared to 1990–1996.

### Implications for future aerosol trends

During 1980–1984, SO_2_ from foreign emissions accounted for 30% of DJF sulfate concentration over the North China Plain (Supplementary Fig. S5). Foreign emission contributions to sulfate decreased to 8% during 2010–2014 due to large decreases of contributions from Russia/Belarus/Ukraine (from 12% to 2%), Europe (from 6% to 1%) and Central Asia (from 7% to 1%) relative to a rapid increase from China (from 70% to 92%). The decreases in foreign emission contribution to sulfate produced a smaller foreign emission contribution to PM_2.5_ in 2010–2014 (3%) compared to 1980–1984 (9%). Given their present small contribution, changes in foreign emissions appear unlikely to have a strong influence on decadal DJF aerosol trends over the North China Plain in the future.

We make approximate estimates of future impacts on the North China Plain pollution using Representative Concentration Pathway 4.5 (RCP4.5, moderate emission)^37^ and 8.5 (RCP8.5, high emission)^38^ scenarios extracted from the gridded datasets. DJF mean PM_2.5_ concentrations are predicted by multiplying the future emissions by present-day aerosol concentration efficiencies (average of 1980–2014) in Fig. 4c,d. The estimates indicate that DJF PM_2.5_ concentrations in the North China Plain will be reduced by more than 50% around year 2040 under both the RCP4.5 and RCP8.5 scenarios due to strong reduction in the North China Plain local emissions, with RCP4.5 producing larger decreasing trend of PM_2.5_ concentration in 2020–2030 (−8.3 μg m^−3^ decade^−1^), compared to RCP8.5 (−4.3 μg m^−3^ decade^−1^). The RCP4.5 emission scenario apparently not only helps global climate mitigation, but also may improve air quality in the near-term over the North China Plain. Emission reductions in the North China Plain would contribute the most to the decreasing DJF PM_2.5_ concentrations (−4.1~−7.4 μg m^−3^ decade^−1^), followed by reductions from the rest of China (−0.5~−0.8 μg m^−3^ decade^−1^). Future foreign emissions play a smaller role, contributing less than −0.2 μg m^−3^ decade^−1^ to the future decadal variation of wintertime PM_2.5_ concentrations. These estimates suggest that aerosol pollution in the North China Plain could be mitigated by reducing local and other source emissions within China. Further reductions in foreign emissions are not expected to have a large influence on China’s pollution trends. Reducing local emissions is the most certain way to improve future air quality in the North China Plain.

## Discussion

The North China Plain PM_2.5_ concentrations show larger increasing trends in 2000–2014 than 1980–2000 in all the other seasons (Supplementary Fig. S6), although the changes in trends pre- and post-2000 are not as steep as in DJF. By isolating the influence of meteorology using normalized emission contributions, we estimate that foreign emissions also had less effect on 2000–2014 trends than in 1980–2000 in these seasons. Foreign emission changes explain 6–9% of the difference of trends in modeled PM_2.5_ concentrations between 2000–2014 and 1980–2000 in these seasons. It suggests that stabilized foreign emissions uncovered North China Plain aerosol pollution potential in all seasons since 2000.

Southern China and Southwestern China are the other two major polluted regions besides the North China Plain. Wintertime PM_2.5_ concentrations in these two regions also increased faster in 2000–2014 compared to 1980–2000 (Supplementary Fig. S7). North China Plain emissions account for a large amount of PM_2.5_ concentrations over these two polluted regions due to aerosol transport from the North China Plain by northerly winds associated with the East Asian winter monsoon. Local and North China Plain emissions are responsible for most of the larger increasing PM_2.5_ in 2000–2014 compared to 1980–2000 over Southern China and Southwestern China. Due to the faster increase in emissions from South Asia during the most recent decade, the total foreign contribution increases faster in 2000–2014 than 1980–2000, explaining 4–5% of the change in trends of PM_2.5_ concentrations in Southern China and Southwestern China pre- and post-2000. It is interesting that, unlike the pollution enhancement effect by meteorology in the North China Plain, variations in meteorology strongly attenuate the contribution of emission changes to the trend differences between 1980–2000 and 2000–2014 over Southern China, primarily because the weakening of monsoon winds reduced the transport of the North China Plain emissions to Southern China.

Note that, previous studies^31,39^ reported modeled aerosol concentrations were largely underestimated in China with the same model configuration used in this study, and attributed the bias to resolution differences between model grids and observations, uncertainties in gas-particle transformation, too much aerosol removal, and/or uncertainties in both local and remote emissions. The aerosol concentration bias may lead to biases in trends of aerosol concentrations, especially contributed by China emissions. However, the bias will not influence the key point in this study that stabilized foreign emissions uncovered the winter pollution potential in the North China Plain from domestic emission increases. (While the simulation performed in this study is driven by version 2016 of CMIP6 emissions, we also calculated normalized PM_2.5_ concentrations based on updated version 2017 of emissions and aerosol concentration efficiencies from this study (Supplementary Fig. S8), and no discernable difference is evident between these two version of emissions).

In this study, PM_2.5_ is identified as the sum of sulfate, BC and POM without considering SOA, due to the high uncertainties in SOA precursor gas emissions and treatment of gas-to-particle-conversion processes in global models. In our simulation, with SOA included in PM_2.5_, the variation in PM_2.5_ concentration does not show a significant change compared to that with SOA excluded (Supplementary Fig. S9). SOA does not perturb the trend of PM_2.5_ over 1990–1996 (−4.8 ± 2.9 μg m^−3^ decade^−1^ with SOA included versus −4.7 ± 2.8 μg m^−3^ decade^−1^ with SOA excluded), but does contribute to the increasing trend after 1996 (9.5 ± 2.6 μg m^−3^ decade^−1^ versus 8.5 ± 2.4 μg m^−3^ decade^−1^). It suggests that the definition of PM_2.5_ without considering SOA may lead to a slight underestimation of the recent increasing trend of PM_2.5_, but does not change the main conclusions of this study. It should also be noted that previous study found that the SOA can have a similar contribution as secondary inorganic aerosol (e.g. sulfate) to haze in China^1^, indicating that SOA concentrations in China are likely underestimated in the model.

Aerosol pollution in China has worsened during the recent two decades. We have shown that the trend in PM_2.5_ in the North China Plain during 1980–2000 from local emissions was obscured by 13% due to reductions in foreign emissions. As contributions from foreign emissions became smaller and stabilized, their counteracting effect nearly disappeared during 2000–2014, uncovering additional pollution increases from domestic emissions. The slowdown in the impact from foreign emission reductions together with weakening of winds explain about 25% of the increased trend of PM_2.5_ in the 21^st^ century compared to pre-2000. The findings of this study highlight a significant contribution of changes in foreign emissions to historical changes in haze occurrence in the North China Plain that needs to be taken into account in air quality studies.

## Methods

### Inverse of observed atmospheric visibility

The inverse of observed atmospheric visibility, which is closely related to light extinction by particles, is used to evaluate the model’s performance in reproducing decadal variations of wintertime PM_2.5_ concentrations in the North China Plain^23^. In each of the 346 meteorological stations in China, DJF mean visibility is calculated as the average of observed daily visibility in DJF for each year between 1981 and 2013. Rainy days and days with daily mean relative humidity larger than 90% are excluded from the calculation to minimize the influence of humidity changes on visibility^40^. Then a regional mean visibility is calculated as the average of visibility for stations within the North China Plain. The inverse of observed atmospheric visibility is calculated as the reciprocal of the regional mean visibility.

### Model description and simulation

To examine the decadal variation of wintertime aerosols in the North China Plain, a 36-year simulation (1979–2014) has been performed with time-varying insolation, surface conditions (e.g., sea surface temperature and ice concentrations), greenhouse gases and aerosol emissions using the Community Earth System Model (CESM)^41^. Aerosols, including sulfate, black carbon (BC), primary organic matter (POM), second organic aerosol (SOA), mineral dust and sea salt, are simulated in the three-mode modal aerosol module^42^ of the Community Atmosphere Model version 5 (CAM5), which is the atmospheric model in CESM. The simulation was performed with a 1.9° latitude by 2.5° longitude horizontal grid spacing and 30 vertical layers. A set of model modifications has also been included to improve the model performance of aerosol wet scavenging and convective transport^43^. Model wind fields are nudged to the MERRA (Modern Era Retrospective-Analysis for Research and Applications) reanalysis^44^ at 6-hour timescale in order to match observed large-scale circulation patterns. Results for 1980–2014, after one-year model spin-up, are used for analysis. Winter season is defined as December in the previous year and January, February in the examined year. Anthropogenic^45^ and open fire emissions^46^ are from the CMIP6 (Coupled Model Intercomparison Project Phase 6) datasets. PM_2.5_ is identified as the sum of sulfate, BC and POM in this study. Although SOA can also contribute to PM_2.5_, its precursor gas emissions and treatment of formation processes in global models are very uncertain. Thus the SOA contribution to PM_2.5_ is not considered in this study. Aerosol concentrations and trends examined in this study are in the atmospheric layer near the surface.

### Aerosol source tagging method

To quantify the role of China and foreign emissions in the decadal variations of wintertime aerosol pollution in the North China Plain, an aerosol source tagging capability implemented in CAM5^31,39,47^ is used in this study. With this capability, BC, POM, sulfate and precursor gases from independent sources can be explicitly tagged as separate tracers and tracked within one simulation. This technique neither perturbs aerosol emissions nor employs assumptions for aerosol sources and sinks along the transport pathways. Transport, physics and chemistry tendencies are calculated separately for each tagged aerosol and precursor gas in the same way as the original aerosol tracers in CESM. Source emissions from the North China Plain (NTC), rest of China (RCN) (including Southern China (STC), Southwestern China (SWC), Central-Western China (CWC), Northeastern China (NEC), and Himalayas and Tibetan Plateau (HTP)) and foreign emissions from rest of East Asia (REA), South Asia (SAS), Southeast Asia (SEA), Russia/Belarus/Ukraine (RBU), Europe (EUR), the Middle East (MDE), Central Asia (CAS) and rest of the World (ROW) are tagged in this study (Supplementary Fig. S1). The sub-regions of rest of China (RCN) are only used to calculate aerosol concentration efficiency and normalized concentration in the North China Plain. Natural (e.g. oceanic and volcanic) emissions are grouped into ROW emissions. The North China Plain (109°E–east border, 30–41°N) is the receptor region we focus on in this study.

### Aerosol concentration efficiency and normalized concentration

To quantify the relationship between emission and concentration over the North China Plain, aerosol concentration efficiency is calculated for each source region and each aerosol component as:

2 $${F}_{s,y}=\frac{{C}_{s,y}}{{E}_{s,y}}$$

in which *F*, *C* and $E$ represent aerosol concentration efficiency, aerosol concentration contributed by a certain source region, and total emission from that source region, respectively. $s$ and $y$ denote source region and year, respectively. By the definition, aerosol concentration efficiency represents the mean aerosol concentration produced by per unit emission, of which the interannual variation is primarily driven by mixing, transport and removal associated with changes in meteorological conditions. To quantify the influence of variation in emissions from different source regions on the North China Plain aerosol concentrations, the signal of meteorological variation can be removed by normalizing aerosol concentrations using aerosol concentration efficiencies. The normalized aerosol concentration over the North China Plain contributed by a certain source region for a certain year (${N}_{s,{y}}$) is calculated as:

3 $${N}_{s,y}={C}_{s,y}\times \frac{1/n{\sum }_{y=1}^{n}{F}_{s,y}}{{F}_{s,y}}$$

in which $n$ represents the total number of years (35 in this study). $C$ is original modeled aerosol concentration, including sulfate, POM and BC.

### Decomposition of contributions to the difference in PM_2.5_ trends

The larger increasing trend of modeled PM_2.5_ concentration since the beginning of the 21^st^ century (2000–2014), as compared to that in 1980–2000, can be decomposed into contributions from changes in domestic emissions $(\frac{{\rm{\Delta }}T{R}_{DOM}}{{\rm{\Delta }}TR})$, foreign emissions $(\frac{{\rm{\Delta }}{T}{{R}}_{{FOR}}}{{\rm{\Delta }}{TR}})$ and meteorology $(\frac{{\rm{\Delta }}T{R}_{MET}}{{\rm{\Delta }}TR})$ as

4 $$1=\frac{{\rm{\Delta }}T{R}_{DOM}}{{\rm{\Delta }}TR}+\frac{{\rm{\Delta }}T{R}_{FOR}}{{\rm{\Delta }}TR}+\frac{{\rm{\Delta }}T{R}_{MET}}{{\rm{\Delta }}TR}$$

in which Δ*TR*, Δ*TR*_*DOM*_, Δ*TR*_*FOR*_ and Δ*TR*_*MET*_ represent the difference in the total trend of modeled DJF PM_2.5_ concentration in the North China Plain (7.2 μg m^−3^ decade^−1^) between 1980–2000 and 2000–2014, and contributions from domestic emissions, foreign emissions, and meteorology to the total difference, respectively. With the influence of meteorology removed, the normalized PM_2.5_ presents a difference of 5.8 μg m^−3^ decade^−1^ (Δ*TR*_*DOM*_ + Δ*TR*_*FOR*_). Changes in meteorology is responsible for the remaining 1.4 μg m^−3^ decade^−1^ (Δ*TR*_*MET*_). Changes in domestic and foreign emissions contribute 5.4 μg m^−3^ decade^−1^ (Δ*TR*_*DOM*_) and 0.4 μg m^−3^ decade^−1^ (Δ*TR*_*FOR*_), respectively, to the difference in the normalized PM_2.5_ trends (5.8 μg m^−3^ decade^−1^) based on the normalized PM_2.5_ calculation using emissions and aerosol concentration efficiencies for each of the tagged source region.

### Data availability statement

The observed atmospheric visibility data were derived from the National Climatic Data Center Global Summary of Day database, which collected data from 346 meteorological stations in China between 1980 and 2013 (http://www.ncdc.noaa.gov/CDO/cdoselect.cmd?datasetabbv=GSOD&countryabbv=&georegionabbv=). The MERRA (Modern-Era Retrospective Analysis for Research and Applications) reanalysis can be found at https://disc.sci.gsfc.nasa.gov/mdisc/. ECMWF (European Center for Medium Range Weather Forecasts) reanalysis data are obtained from http://apps.ecmwf.int/datasets/data/interim-full-moda/levtype=sfc/. NCEP/NCAR reanalysis data are obtained from https://www.esrl.noaa.gov/psd/data/gridded/data.ncep.reanalysis.html. Model results are available through NERSC upon request.

## Electronic supplementary material

Supplementary Information
