# Supplementary material for: Recent intensification of winter haze in China linked to foreign emissions and meteorology
Source: Sci Rep. 2018 Feb 1;8:2107. doi: 10.1038/s41598-018-20437-7 (PMC5794776; doi:10.1038/s41598-018-20437-7)
Supplement: Supplementary file 1 — Supplementary Information [file 41598_2018_20437_MOESM1_ESM.pdf]

Recent intensification of winter haze in China linked to foreign emissions and meteorology

Yang Yang[1*], Hailong Wang[1*], Steven J. Smith[2], Rudong Zhang[1], Sijia Lou[1], Yun Qian[1], Po-Lun Ma[1], Philip J. Rasch[1]

[1]Atmospheric Science and Global Change Division, Pacific Northwest National Laboratory, Richland, Washington, USA

[2]Joint Global Change Research Institute, Pacific Northwest National Laboratory, College Park, Maryland, USA

*Correspondence to yang.yang@pnnl.gov and hailong.wang@pnnl.gov

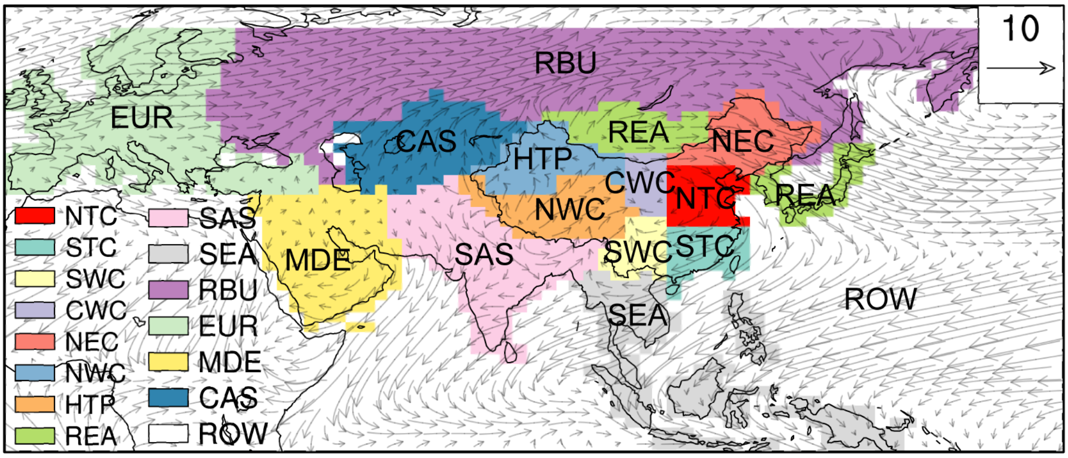

Supplementary Figure S1. Tagged source regions including the North China Plain (NTC), Southern China (STC), Southwestern China (SWC), Central-Western China (CWC), Northeastern China (NEC), Himalayas and Tibetan Plateau (HTP), rest of East Asia (REA), South Asia (SAS), Southeast Asia (SEA), Russia/Belarus/Ukraine (RBU), Europe (EUR), the Middle East (MDE), Central Asia (CAS) and rest of the World (ROW). Vectors represent 925 hPa wind fields (m s^{-1}) in DJF averaged over 1980–2014. Map was generated by NCAR Command Language (NCL) version 6.4.0 (Boulder, Colorado: UCAR/NCAR/CISL/TDD, http://dx.doi.org/10.5065/D6WD3XH5).

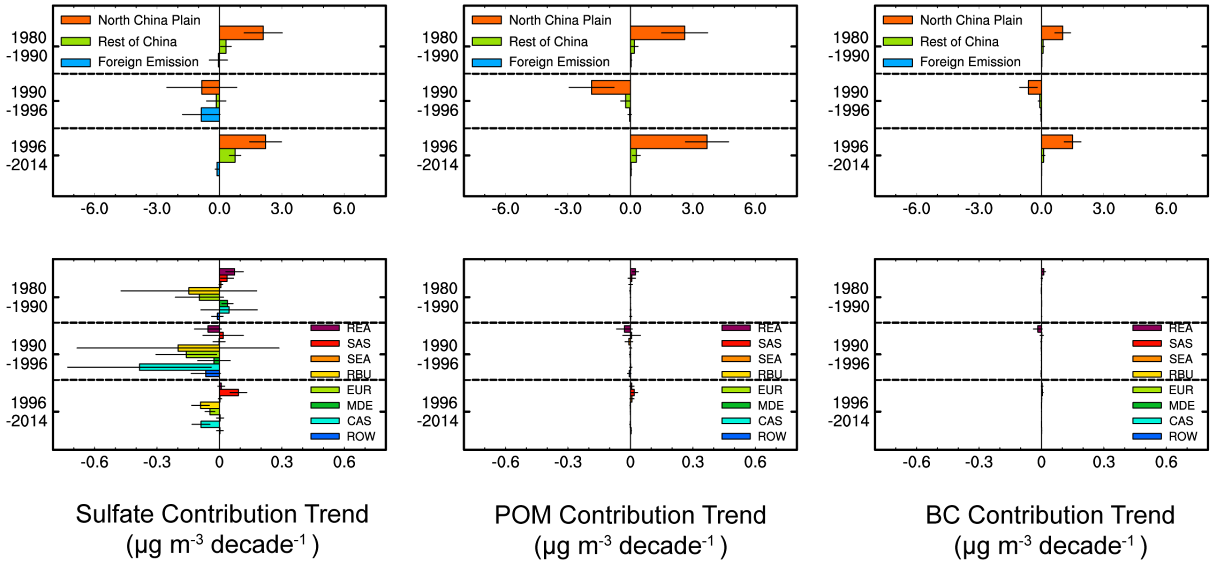

Supplementary Figure S2. Linear trends (µg m^{-3} $decade^{-1}$) of the model simulated DJF mean SO_4 (left), POM (middle) and BC (right) concentrations averaged over the North China Plain contributed by the North China Plain, rest of China and foreign emissions (top) for three time periods, and decomposed foreign contributions by tagged source regions (bottom). Black lines on the bars represent 95% confidence intervals of the linear regression.

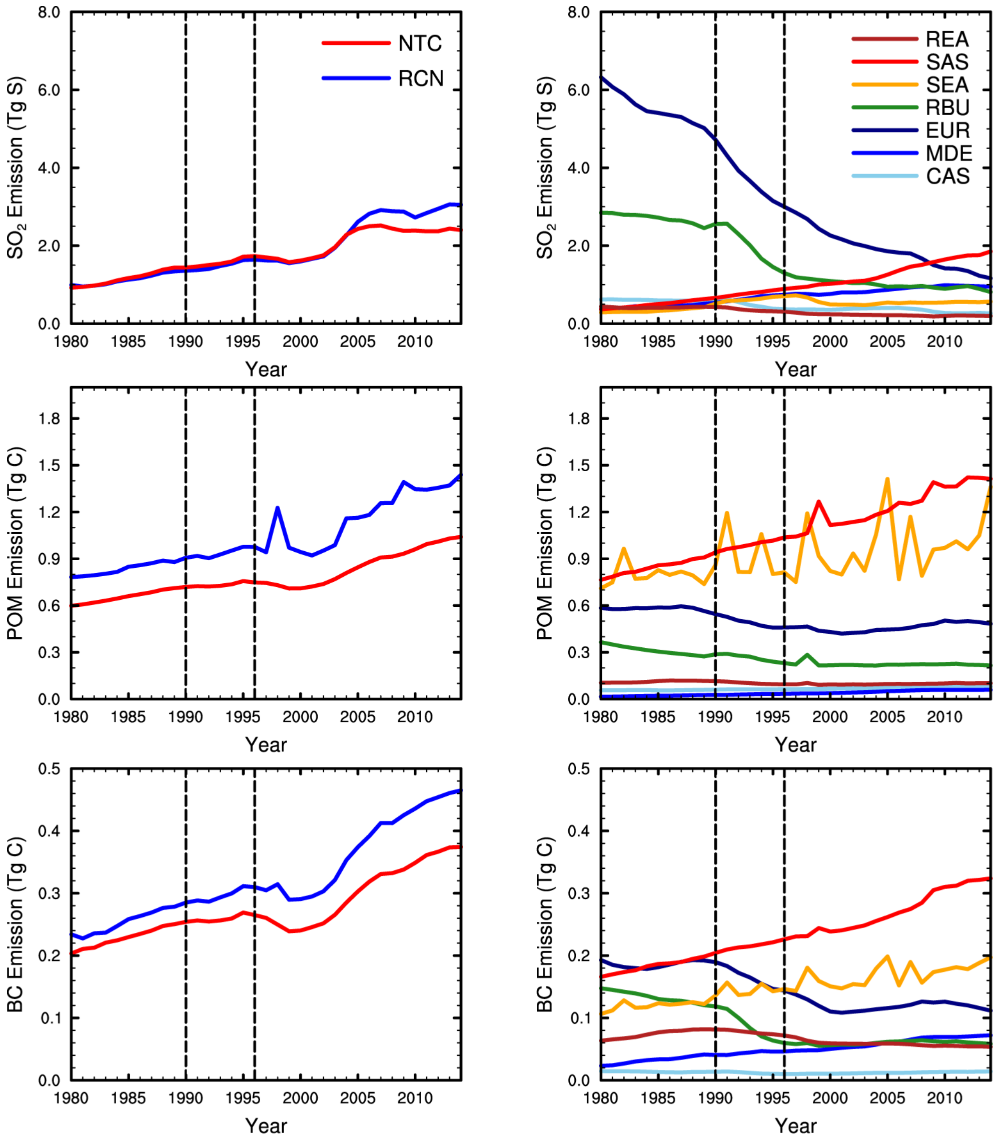

Supplementary Figure S3. Time series (1980–2014) of DJF total SO_2 (top, Tg S), POM (middle, Tg C) and BC (bottom, Tg C) emissions from the North China Plain (NTC, left, red lines), Rest of China (RCN, left, blue lines), and foreign emissions (right). Emissions are from the CMIP6 datasets.

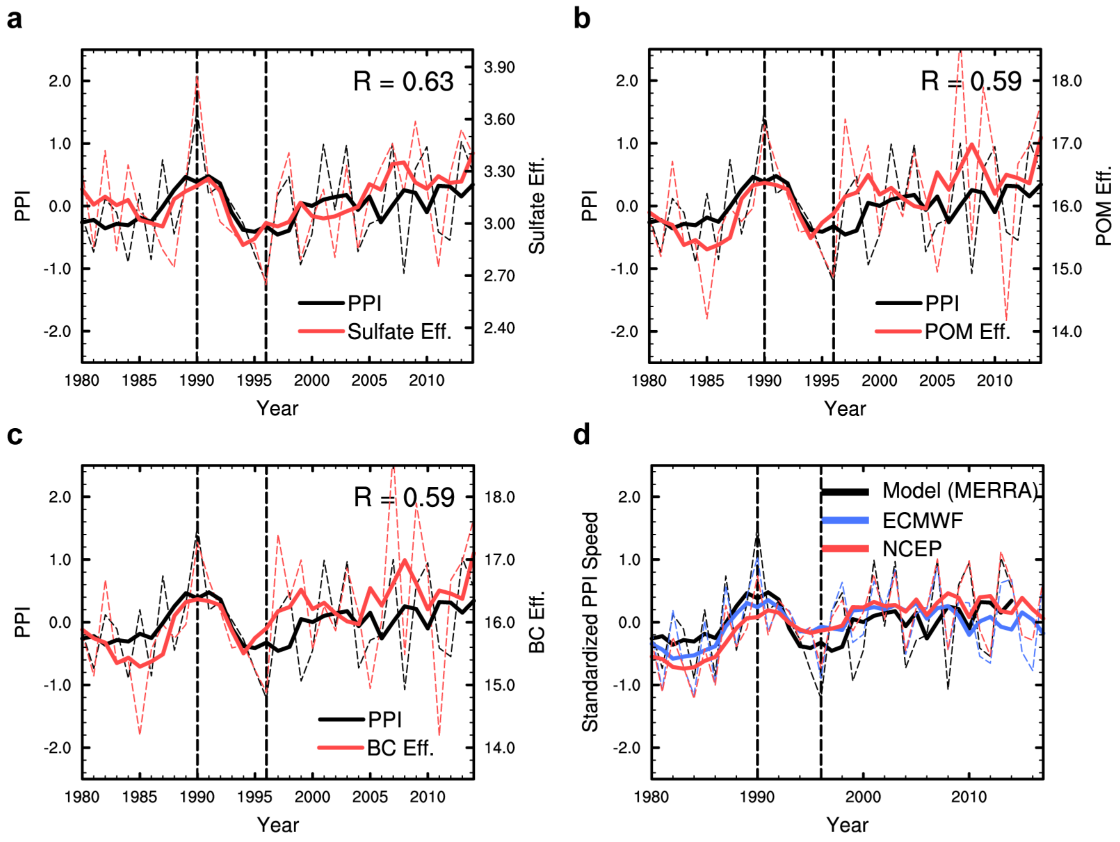

Supplementary Figure S4. Time series of pollution potential index (PPI, black dashed lines) and local concentration efficiency of (a) sulfate, (b) POM and (c) BC (red dashed lines, μg m^{-3} Tg S^{-1} for sulfate and μg m^{-3} Tg C^{-1} for POM and BC) for the North China Plain emissions. (d) PPI in this study, derived from ECMWF reanalysis and NCEP/NCAR reanalysis data. PPI is an index combined regional wind speed and temperature inversion calculated based on Zou et al. (2017). Solid lines represent five-year moving averages. Temporal correlation coefficient (R) between aerosol concentration efficiency and standardized wind speed is shown at the top-right corner of panel a, b and c.

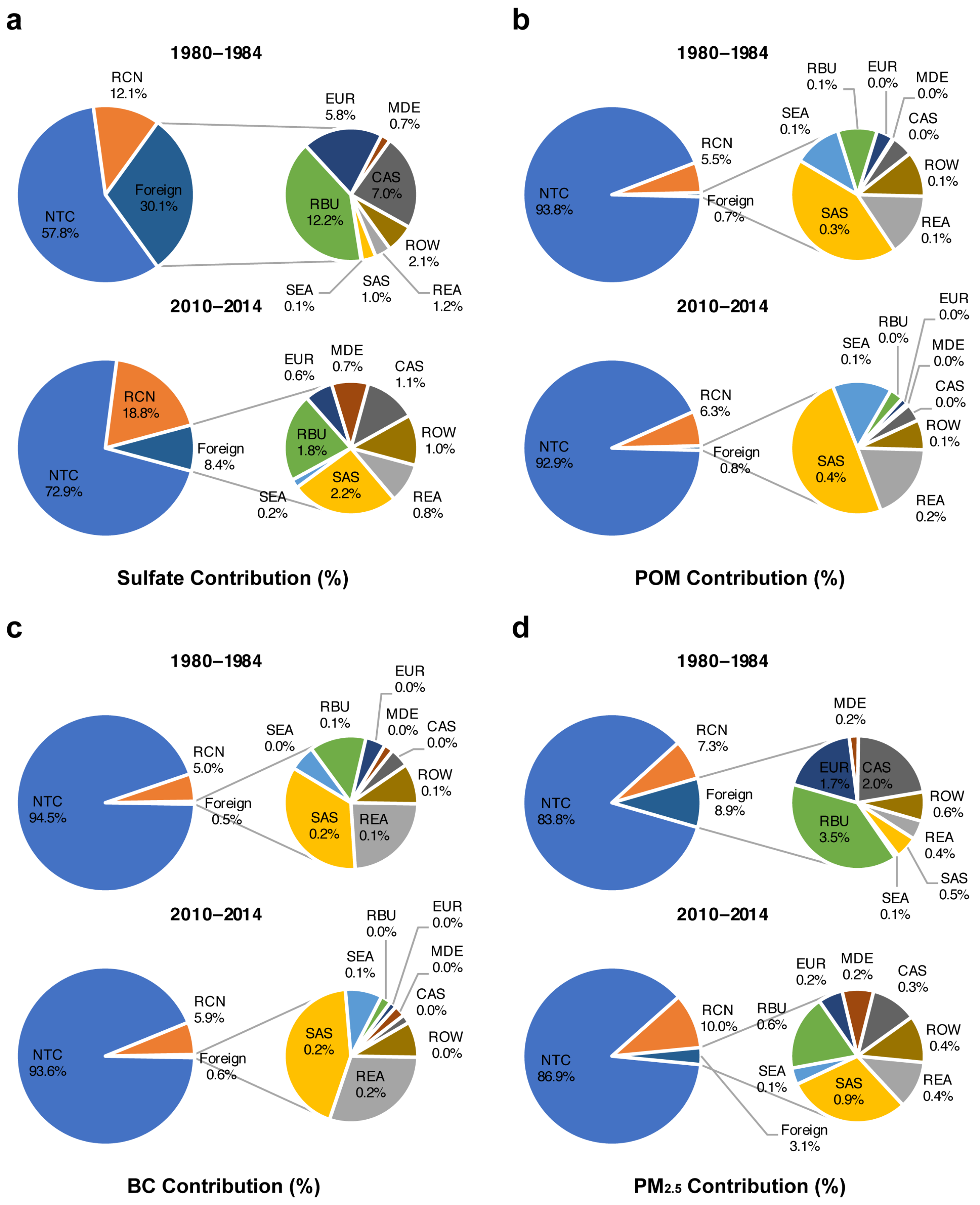

Supplementary Figure S5. Percentage (%) of DJF and regional mean SO_4, POM, BC and $PM_{2.5}$ contributed by the North China Plain, rest of China and foreign emissions, as well as emissions from finer foreign source regions, averaged over 1980–1984 and 2010–2014.

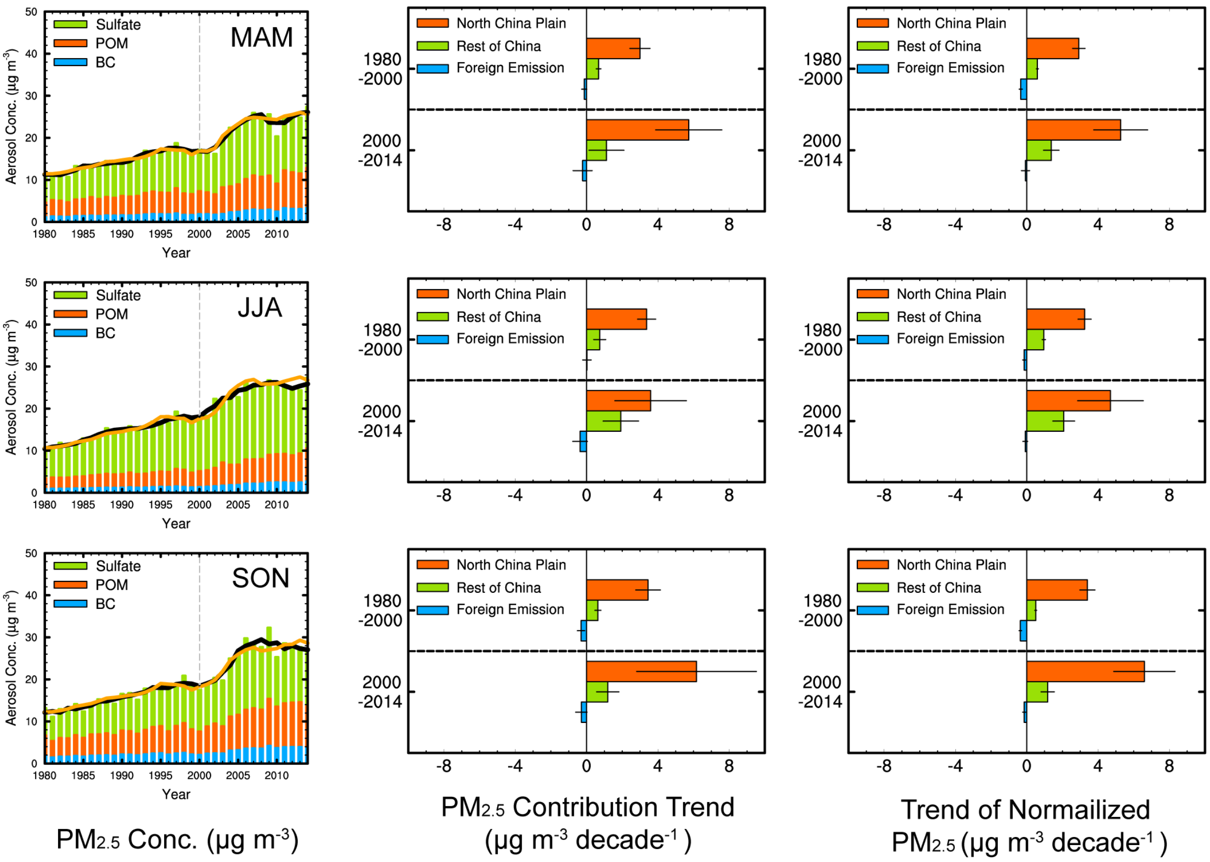

Supplementary Figure S6. Time series of model simulated aerosol concentrations (bars, µg m^{-3}) averaged over the North China Plain (left), linear trends (µg m^{-3} decade^{-1}) of simulated (middle) and normalized (right) $PM_{2.5}$ concentrations contributed by the North China Plain, rest of China and foreign emissions over 1980–2000 and 2000–2014 in March-April-May (MAM), June-July-August (JJA) and September-October-November (SON) (from top to bottom row). Solid black lines in left panels represent five-year moving average. Orange line in left panels represents normalized $PM_{2.5}$ concentrations. Black lines on color bars in middle and right panels represent 95% confidence intervals of the linear regression.

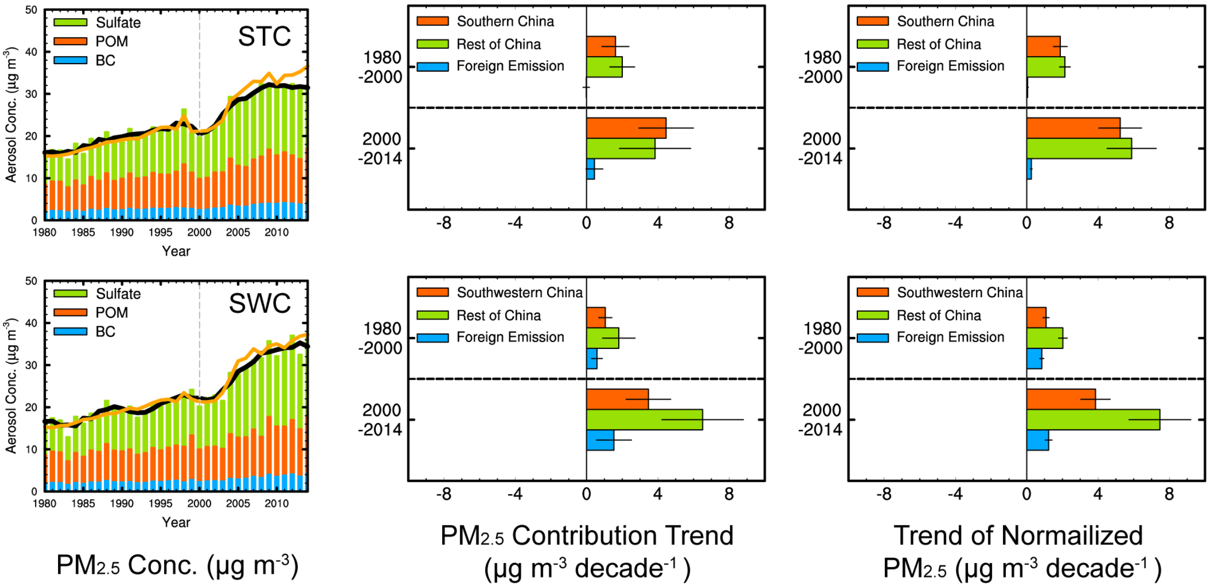

Supplementary Figure S7. Time series of simulated DJF mean $PM_{2.5}$ concentration (bars, µg m^{-3}) (left), linear trends (µg m^{-3} decade^{-1}) of simulated (middle) and normalized (right) $PM_{2.5}$ concentrations in Southern China (top) and Southwestern China (bottom) contributed by local emissions, rest of China and foreign emissions over 1980–2000 and 2000–2014. Orange line in left panels represents normalized $PM_{2.5}$ concentrations. Solid black lines in left panels represent five-year moving average. Black lines on color bars in the middle and right panels represent 95% confidence intervals of the linear regression.

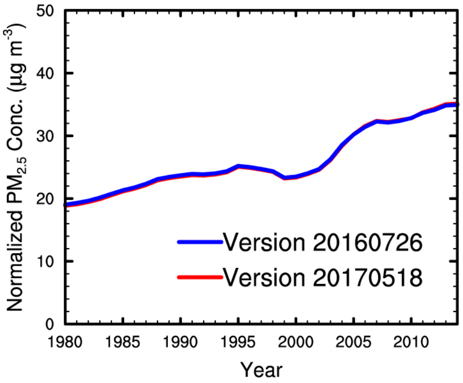

Supplementary Figure S8. Time series of normalized DJF mean $PM_{2.5}$ concentration calculated using version 20160726 (blue line) and 20170518 (red line) of CMIP6 anthropogenic emissions.

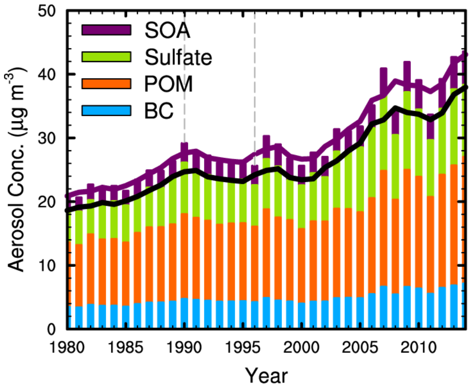

Supplementary Figure S9. Time series of simulated DJF near-surface aerosol concentration (bars) averaged over the North China Plain. Black and purple lines represent five-year moving average of sulfate-POM-BC and sulfate-POM-BC-SOA concentrations, respectively.
